# Comprehensive Description of Pathogens and Antibiotic Treatment Guidance in Children With Community-Acquired Pneumonia Using Combined Mass Spectrometry Methods

**DOI:** 10.3389/fcimb.2021.695134

**Published:** 2021-07-21

**Authors:** Liying Sun, Chi Zhang, Shuhua An, Xiangpeng Chen, Yamei Li, Leshan Xiu, Baoping Xu, Zhengde Xie, Junping Peng

**Affiliations:** ^1^ NHC Key Laboratory of Systems Biology of Pathogens, Institute of Pathogen Biology, Chinese Academy of Medical Sciences & Peking Union Medical College, Beijing, China; ^2^ Key Laboratory of Respiratory Disease Pathogenomics, Chinese Academy of Medical Sciences and Peking Union Medical College, Beijing, China; ^3^ Department of Respiratory Medicine, Hebei Children’s Hospital, Hebei Medical University, Shijiazhuang, China; ^4^ Beijing Key Laboratory of Pediatric Respiratory Infection Diseases, Key Laboratory of Major Diseases in Children, Ministry of Education, National Clinical Research Center for Respiratory Diseases, Research Unit of Critical Infection in Children, Chinese Academy of Medical Sciences, 2019RU016, Laboratory of Infection and Virology, Beijing Pediatric Research Institute, Beijing Children’s Hospital, Capital Medical University, National Center for Children’s Health, Beijing, China; ^5^ National Clinical Research Center for Respiratory Diseases, Research Unit of Critical Infection in Children, Chinese Academy of Medical Sciences, 2019RU016, Respiratory Department, Beijing Children’s Hospital, Capital Medical University, National Center for Children’s Health, Beijing, China

**Keywords:** community-acquired pneumonia, respiratory pathogens, molecular testing, mass spectrometry, antibiotic therapy

## Abstract

The objective of this study was to evaluate the value of molecular methods in the management of community-acquired pneumonia (CAP) in children. Previously developed mass spectrometry (MS)-based methods combined with quantitative real-time PCR (combined-MS methods) were used to describe the aetiology and evaluate antibiotic therapy in the enrolled children. Sputum collected from 302 children hospitalized with CAP were analyzed using the combined-MS methods, which can detect 19 viruses and 12 bacteria related to CAP. Based on the results, appropriate antibiotics were determined using national guidelines and compared with the initial empirical therapies. Respiratory pathogens were identified in 84.4% of the patients (255/302). Co-infection was the predominant infection pattern (51.7%, 156/302) and was primarily a bacterial-viral mixed infection (36.8%, 111/302). Compared with that using culture-based methods, the identification rate for bacteria using the combined-MS methods (61.8%, 126/204) increased by 28.5% (*p <*0.001). Based on the results of the combined-MS methods, the initial antibiotic treatment of 235 patients was not optimal, which mostly required switching to β-lactam/β-lactamase inhibitor combinations or reducing unnecessary macrolide treatments. Moreover, using the combined-MS methods to guide antibiotic therapy showed potential to decrease the length of stay in children with severe CAP. For children with CAP, quantitative molecular testing on sputum can serve as an important complement to traditional culture methods. Early aetiology elucidated using molecular testing can help guide the antibiotic therapy.

## Highlights

1) We used combined-MS methods to comprehensively analyze the etiology in children with CAP in North China;
2) Early etiology obtained using molecular testing in our study can be introduced to guide antibiotic therapy;
3) We provided valuable reference for the principles and practices of antibiotic stewardship in children hospitalized for CAP.

## Introduction

Pneumonia is the predominant cause of infection-related deaths in young children, with approximately 0.9 million deaths annually in children younger than 5 years of age (Bryce et al., 2005, Liu et al., 2016). The infectious agents of community-acquired pneumonia (CAP) are diverse. Currently, clinical microbiology laboratories rely heavily on sputum culture; however, culture-based methods are time-consuming and less sensitive, which makes them less useful for timely diagnosis. Based solely on the results of culture, a precise microbiological diagnosis can be made in less than 15% of children hospitalized with CAP (Bradley et al., 2011). Owing to the limitations of culture-based methods, empirical antimicrobials are administered to patients who do not have a precise microbiological diagnosis. Such untargeted antibiotic selection or excessive use of antimicrobials is associated with increased antimicrobial resistance (Duong et al., 2018).

To comprehensively and rapidly identify the causative agents of CAP, new methods are urgently needed. Compared with traditional culture-based methods, molecular testing offers the following advantages: besides high sensitivity and shorter turnaround time, it has the superiority of detecting multiple pathogens, which can substantially improve identification efficiency. Molecular-based tests are also crucial for identifying respiratory viruses and atypical pathogens that require rigorous culture conditions, like *Legionella pneumophila*, *Bordetella pertussis*, *Mycoplasma pneumoniae*, and *Chlamydophila pneumoniae*. Currently, the most frequently used molecular-based method is real-time PCR; however, it can only detect a limited number of pathogens simultaneously (Caliendo, 2011). Therefore, when screening diverse candidate pathogens of CAP, multiple reactions are needed, which is time-consuming and labor intensive. To perform multiple reactions, a larger sample size is required, placing an additional burden on patients, especially children. To overcome these limitations, we developed two multiplex PCR coupled with matrix-assisted laser desorption ionisation-time of flight mass spectrometry (MALDI-TOF MS), common respiratory virus-mass spectrometry (CRV-MS), and bacterial pathogen-mass spectrometry (BP-MS).

The viral pathogen panel (CRV-MS) can simultaneously detect and identify 19 common respiratory virus types/subtypes, and the bacterial panel (BP-MS) can screen 12 bacterial pathogens associated with CAP (Zhang et al., 2015; Zhang et al., 2018). The CRV-MS and the BP-MS methods are therefore suitable for large-scale pathogen screening and epidemiological studies.

Here, we used the CRV-MS and BP-MS methods to detect pathogens in sputum from children hospitalized with CAP retrospectively. By comparing the results with those of the culture-based method, we evaluated the potential of combined-MS in improving the detection rate of pathogens and to guide initial antimicrobial therapy. Moreover, we described the aetiology of CAP in North China by analysing the results of combined-MS methods.

## Materials and Methods

### Subjects

**Figure 1:**
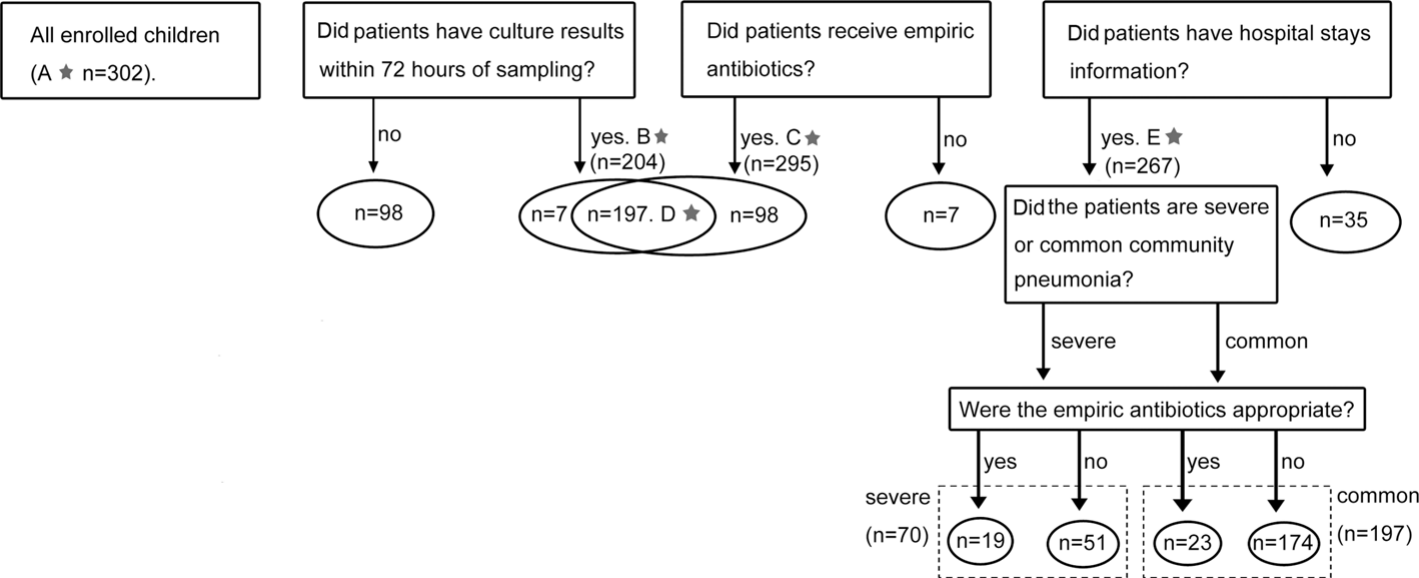
Cases enrolment and study design. The numbers marked with a star referred to the sample size of five groups. **(A)** Results of combined-MS methods obtained from all 302 patients were used to define the etiology of CAP. **(B)** 204 cases with culture results within 72 hours of sampling were used to compare the performance of combined-MS methods and culture. **(C)** 295 cases with information of empirical antimicrobial therapy were used to study the value of combined-MS methods in guiding antimicrobial prescribing. **(D)** 197 cases with both information of culture and empirical antimicrobial therapy were used to investigate the impact of antibiotic usage on the performance of combined-MS methods and culture. **(E)** 267 patients with information of LOS were used to assess how antibiotic therapy affect LOS. LOS, length of stay.

A total of 302 children hospitalized with CAP between January 2016 and December 2018 were included in this study. They were all younger than 16 and admitted to Beijing Children’s Hospital (Beijing, China) and Children’s Hospital of Hebei Province (Shijiazhuang, Hebei Province, China). CAP was diagnosed according to the Chinese national guidelines for the management of CAP in children, update 2013. All children had fever (body temperature ≥ 38°C), cough, tachypnoea, dyspnoea, chest retractions, abnormal auscultatory findings, and radiological evidence of CAP. The severity of CAP was also evaluated based on the same guidelines. Patient information on age, sex, length of stay (LOS), sputum culture results, empirical antibiotic prescription, or antibiotic administration before hospitalisation were retrospectively collected from electronic medical records. All enrolled patients were divided into five overlapping groups for different study aims (**Figure 1**). For sputum culture, only results obtained within 72 hours of the sampling time were included in subsequent analyses.

### Specimen Collection and Nucleic Acid Extraction

Sputum was collected within 72 hours of patient admission. Nebulisation was used for younger children. Induced sputum samples from the inhalation of hypertonic saline solution were collected by trained personnel according to standard operating procedures, as published previously (Lahti et al., 2009; Honkinen et al., 2012).

A total of 2 mL of sputum was obtained from each patient with sterility sputum aspiratory tubes and stored at -80°C. The nucleic acids of the viruses were extracted using QIAamp MinElute Virus Spin kits (QIAGEN, Hilden, Germany), and cDNA was synthesized using SuperScript First-Strand Synthesis System (Invitrogen, Carlsbad, CA, USA) according to the manufacturer’s instructions. To extract bacterial DNA, 200 μL of sputum were centrifuged at 5000 × g for 10 min, and pellets resuspended in 150 μL enzyme cocktail containing 6 mg lysozyme, 30 U lysostaphin, 37.5 U mutanolysin, and 30U lyticase in lysis buffer of 20 mM Tris-HCl (pH 8), 2 mM EDTA, and 1.2% Triton. The mixture was incubated at 37°C for 30 min to lyse the cell walls of Gram-positive bacteria. DNA was then purified using the QIAamp DNA Mini Kit (Qiagen, Hilden, Germany) according to the manufacturer’s instructions. Purified DNA was eluted in 200 μL nuclease-free water.

### Cultural and Combined-MS Methods

Sputum culture and identification were performed using standard microbiological and biochemical methods.

The CRV-MS method can simultaneously identify 19 common respiratory viruses, including adenovirus (AdV), human enterovirus (EV), four human coronaviruses (HCoV-OC43, 229E, NL63, and HKU1), human bocavirus 1 (HBoV1), human metapneumoviruses A and B (HMPV-A and HMPV-B), human rhinovirus (HRV), influenza A viruses (IFV-A H1N1, and H3N2), influenza B viruses (IFV-B), parainfluenza virus types 1–4 (PIV1 to-4), and respiratory syncytial viruses A and B (RSV-A and RSV-B, respectively) (Jain, 2017). The BP-MS method can simultaneously identify 12 bacterial pathogens related to pneumonia, including *Legionella pneumophila*, *Bordetella pertussis*, *Mycoplasma pneumoniae*, *Chlamydophila pneumoniae*, *Haemophilus influenzae*, *Staphylococcus aureus*, *Moraxella catarrhalis*, *Klebsiella pneumoniae*, *Pseudomonas aeruginosa*, *Acinetobacter baumannii*, *Streptococcus pneumoniae*, and *Escherichia coli*. Detailed procedures for CRV-MS and BP-MS have been described previously (Zhang et al., 2015; Zhang et al., 2018).

Among the bacterial targets, *H. influenzae*, *S. aureus*, *M. catarrhalis*, *K. pneumoniae*, *P. aeruginosa*, *A. baumannii*, *S. pneumoniae*, and *E. coli* are commonly detected commensals of the respiratory tract, which may act as contaminants of the sputum (Apisarnthanarak and Mundy, 2005; Jain, 2017). As BP-MS is a qualitative method, positive results for the above bacteria were retested by quantitative real-time PCR to obtain their bacterial loads. Only bacteria with a load of ≥10^5^ CFU/mL can be regarded as a pathogen responsible for infection (combined-MS methods positive) (Gadsby et al., 2016). The assays used in the real-time methods refer to previous studies (Zhang et al., 2018).

### Antibiotics Selected for Pathogen-Guided Therapy

According to the results of combined-MS methods, the right antibiotics were determined using the Children’s Community Pneumonia Diagnosis and Treatment Guidelines (2013 revised) and revised World Health Organization (WHO) Classification and Treatment of Pneumonia in Children at Health Facilities: Evidence Summaries (see **Supplementary Material**, **Table S1**) (Subspecialty Group of Respiratory Diseases and Chinese Medical Association The Editorial Board, 2013; WHO, 2014).

### Statistical Analysis

Categorical data were compared using the chi-squared (χ^2^) or Fisher’s exact tests. Paired proportions were compared using the McNemar test. Continuous variables were analyzed using the Shapiro-Wilk test for normality. Student’s t-test and the Mann-Whitney U test were used to compare normally and non-normally distributed continuous variables, respectively. The above statistical analyses were performed with SPSS (Statistical Product and Service Solutions) software version 19, and a *P* value <0.05 was considered statistically significant.

## Results

### General Characteristics of Patients Included

Among 302 included children, the median age was 1 year (range, 0.04-16 years), and 65.9% (199) were male (**Table 1**). Children were categorised into neonates (age < 1, 47.0%, 142), toddlers (1 ≤ age < 3, 23.9%, 72), pre-schoolers (3 ≤ age < 6, 8.6%, 26), and school-age children (6 ≤ age ≤ 16, 20.5%, 62, **Table 1**). Patients were divided into four groups based on sampling times: spring (26.5%, 80), summer (4.6%, 14), autumn (5.6%, 17), and winter (63.3%, 191).

**Table 1:** Characteristics of 302 included children.

Characteristics	N (%)
Sex	
Male	199 (65.9)
Female	103 (34.1)
Age (yr)	
Age < 1 (neonatal group)	142 (47.0)
1 ≤ age < 3 (toddler group)	72 (23.9)
3 ≤ age < 6 (preschool group)	26 (8.6)
6 ≤ age < 16 (school group)	62 (20.5)
Sampling time	
Spring (from March to May)	80 (26.5)
Summer (from June to August)	14 (4.6)
Autumn (from September to November)	17 (5.6)
Winter (from December to February)	191 (63.3)
Outcome	
Discharge	299 (99.01)
Death	3 (0.99)

### Aetiology of CAP in Hospitalised Children Identified by Combined-MS Methods

Respiratory pathogens were identified in 84.4% of patients (255). Among them, 64.9% (196) were bacteria and 56.3% (170) were virus-positive. Specifically, RSV was the most common pathogen (26.5%, 80), followed by *H. influenzae* (22.2%, 67), *S. pneumoniae* (20.5%, 62), and *M. pneumoniae* (14.9%, 45, **Table 2**).

**Table 2:** Detection rate of common respiratory pathogens with combined-MS methods (n = 302).

Pathogens	N (%)
Any pathogen	255 (84.4)
Any bacteria	196 (64.9)
* L. pneumophila*	3 (1.0)
* B. pertussis*	10 (3.3)
* M. pneumoniae*	45 (14.9)
* C. pneumonia*	1 (0.3)
* S. pneumoniae*	62 (20.5)
* H. influenzae*	67 (22.2)
* S. aureus*	36 (11.9)
* M. catarrhalis*	25 (8.3)
* P. aeruginosa*	6 (2.0)
* A. baumannii*	7 (2.3)
* K. pneumoniae*	9 (3.0)
* E. coli*	10 (3.3)
Any virus	170 (56.3)
Human coronavirus (HCoV)	8 (2.7)
HCoV-229E	5 (1.7)
HCoV-HKU1	1 (0.3)
HCoV-NL63	0 (0)
HCoV-OC43	2 (0.7)
Adenovirus (AdV)	30 (9.9)
Enteroviruses (EV)	31 (10.3)
Human bocavirus (HBoV)	13 (4.3)
Human metapneumovirus (HMPV)	7 (2.3)
HMPV-A	6 (2.0)
HMPV-B	1 (0.3)
Human rhinovirus (HRV)	27 (8.9)
Influenza (IFV)	27 (8.9)
IFV-A-H1	13 (4.3)
IFV-A-H3	2 (0.7)
IFV-B	12 (4.0)
Parainfluenza virus (PIV)	12 (4.0)
PIV-1	1 (0.3)
PIV-2	0 (0)
PIV-3	11 (3.6)
PIV-4	0 (0)
Respiratory syncytial virus (RSV)	80 (26.5)
RSV-A	50 (16.6)
RSV-B	30 (9.9)

**Figure 2:**
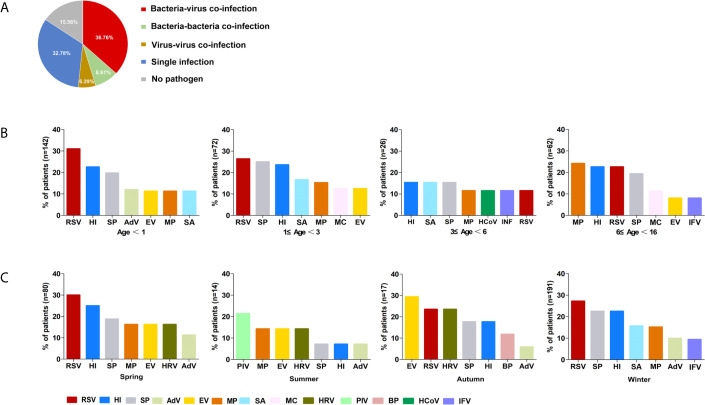
The etiology of 302 included children defined by combined-MS methods. **(A)** The proportions of different patterns of infection. **(B)** Distribution of main pathogens in patients with different age groups. **(C)** Seasonal prevalence of main pathogens. RSV, respiratory syncytial virus; HI, *Haemophilus influenzae*; SP, *Streptococcus pneumoniae*; MP, *Mycoplasma pneumoniae*; SA, *Staphylococcus aureus*; EV, Enterovirus; AdV, Adenovirus; IFV, Influenza virus; HRV, Human rhinovirus; MC, *Moraxella catarrhalis*; PIV, Parainfluenza virus; BP, *Bordetella pertussis*; HCoV, Human coronavirus.

Mixed infection was the most frequent pattern in patients (51.7%, 156). Of 156 mixed infection cases, dual pathogen co-infection (57.0%, 89) was the most common, followed by triple (26.3%, 41), quadruple (11.5%, 18), and mixed-infection with more than four pathogens (5.1%, 8). Bacterial-viral mixed infection (36.8%, 111/302) was the primary co-infection pattern. *H. influenzae/*RSV (12.8%, 20/156) was the most common co-infection combination, followed by *S. pneumoniae*/*H. influenzae* (11.5%, 18/156) and *S. pneumoniae/*RSV (11.5%, 18/156). No pathogen was detected in 47 (15.6%, 47/302) specimens (**Figure 2**).

The predominant respiratory pathogens in children with CAP varied with age. RSV was the most frequently detected pathogen in children under age 3 (29.4%, 63/214), *H. influenzae* was found in children aged 3 to 6 (15.4%, 4/26), and *M. pneumoniae* was found in children aged 6 to 16 (24.2%, 15/62; **Figure 2B**). The primary pathogens changed with seasons. RSV was most commonly identified in both spring and winter (30%, 24/80; 27%, 52/191) and PIV and EV were most frequently detected in summer (21.4%, 3/14), and autumn (29.4%, 5/17), respectively. (**Figure 2C**). However, there was no statistically significant difference in mixed infection rates among the age groups or season (*P* > 0.05).

### Comparison of Performance Between Culture-Based and Combined-MS Methods

The detection rate of ten culturable bacterial pathogens using combined-MS methods was significantly higher than that using sputum culture (61.8%, 126 versus 33.3%, 68, *P* < 0.001, **Table 3**) in 204 patients with culture-based detection information. Compared with culture-based methods, combined-MS methods detected more of the following pathogens: *S. pneumoniae* (6.9% vs. 20.6%, *P* < 0.001), *H. influenzae* (6.9% versus 23.1%, *P* < 0.001), *S. aureus* (6.9% versus 16.2%, *P* < 0.001), and *M. catarrhalis* (2.5% versus 9.9%, *P* < 0.001, **Table 3**). For the other four pathogens, the detection rate of combined-MS methods was higher than that of culture-based methods, but the difference was not statistically significantly (**Table 3**, *P* > 0.05). In the other ten patients, bacteria not selected as targets of combined-MS methods were detected using culture-based methods (see **Supplementary Material**, **Table S2**).

**Table 3:** Comparison of the performance between combined-MS methods and culture-based methods (n = 204).

	Combined-MS methods	Culture-based methods		*P ^b^*
		Positive	Negative	
		n (%) ^c^	n (%)	
Ten culturable pathogens ^a^	Positive	63 (30.9)	63 (30.9)	*P* <0.001
	Negative	5 (2.4)	73 (35.8)	
*S. pneumoniae*	Positive	14 (6.9)	28 (13.7)	*P* <*0.001*
	Negative	0 (0)	162 (79.4)	
*H. influenzae*	Positive	13 (6.4)	34 (16.7)	*P* <*0.001*
	Negative	1 (0.5)	156 (76.5)	
*S. aureus*	Positive	12 (5.9)	21 (10.3)	*P* <*0.001*
	Negative	2 (1.0)	169 (82.8)	
*M. catarrhalis*	Positive	5 (2.5)	15 (7.4)	*P* <*0.001*
	Negative	0 (0)	184 (90.2)	
*P. aeruginosa*	Positive	4 (2.0)	1 (0.5)	*P* >*0.05*
	Negative	1 (0.5)	198 (97)	
*A. baumannii*	Positive	3 (1.5)	2 (1)	*P* >*0.05*
	Negative	2 (1)	197 (96.5)	
*K. pneumoniae*	Positive	4 (2.0)	3 (1.4)	*P* >*0.05*
	Negative	2 (1.0)	195 (95.6)	
*E. coli*	Positive	3 (1.5)	3 (1.5)	*P* >*0.05*
	Negative	1 (0.5)	197 (96.5)	
*L. pneumophila*	Positive	0 (0)	2 (1)	*P* >*0.05*
	Negative	0 (0)	202 (99.0)	
*B. pertussis*	Positive	0 (0)	2 (1)	*P* >*0.05*
	Negative	0 (0)	202 (99.0)	

^a^Ten culturable pathogens included S. pneumoniae, H. influenzae, S. aureus, M. catarrhalis, P. aeruginosa, A. baumannii, K. pneumoniae, E. coli, L. pneumophila, and B. pertussis. ^b^P values were calculated using the McNemar test. ^c^The number and the percentage.

### Impact of Antibiotic Use on the Performance of Combined-MS Methods and Culture

Among 197 cases who received empirical antimicrobial therapy, the detection rate of ten culturable pathogens using combined-MS method was 60.9% (120), which was higher than the rate using culture (29.4%, 58*, P* < 0.001). The results showed that compared with culture, the impact of initial empirical antibiotic usage on bacterial detection was lower in combined-MS methods.

### Using the Results of the Combined-MS Methods to Evaluate Initial Empirical Antibiotic Treatment

In 295 patients who received empirical antimicrobial therapy based on to the results of combined-MS methods, the initial antibiotic therapies of 235 patients were not optimal. Most class of antibiotics (39.0%, 115) needed to change and the number of antibiotics needed to decrease (36.9%, 109, **Table 4**). Changing the class of antibiotics mainly involved switching β-lactam (26.1%, 77) or macrolide antibiotics (8.5%, 25) to a β-lactam/β-lactamase inhibitor combinations. Decreasing the number of excessive antibiotics mainly referred to a reduction in the use of macrolide antibiotics (25.4%, 75) and β-lactam agents (10.2%, 30). The number of antibiotics needed to be increased in 11 patients (3.8%, 11) while 60 patients (20.3%, 60) needed no change.

**Table 4:** Using the results of combined-MS methods to guide the antibiotic treatment (n = 295).

Antibiotics modify	N (%)
Change class	115 (39.0)
LA to LIC	77 (26.1)
MA to LIC	25 (8.5)
LIC to LA	4 (1.4)
MA to LA	3 (1.0)
LA to MA	3 (1.0)
LIC to MA	3 (1.0)
Decrease numbers	109 (36.9)
MA	75 (25.4)
LA	30 (10.2)
LIC	1 (0.3)
LIC+MA	2 (0.7)
LA+MA	1 (0.3)
Increase numbers	11 (3.8)
LIC	4 (1.4)
MA	3 (1.0)
Increase numbers and change class	
LA to LIC+MA	4 (1.4)
No change	60 (20.3)

LA, β-lactam; LIC, β-lactam/β-lactamase inhibitor combinations; MA, macrolides.

### Assessment of LOS Affected by Antibiotic Therapy

Among 70 children with severe CAP, the mean LOS was significantly shorter for children who did not need to change antibiotic than those who needed therapy change (11.63 ± 0.95 days vs. 18.57 ± 1.97 days, *P* = 0.037). Additionally, in children with common CAP, the mean LOS was also shorter for those not needing therapy change than those who needed a change, though the difference was not statistically significant (11.83 ± 1.34 days versus 13.38 ± 2.20 days, *P* = 0.79).

## Discussion

This study demonstrated that molecular methods to identify infectious pathogens in children with CAP can significantly increase detection rates and molecular methods have the potential to guide antimicrobial selection. Our findings were consistent with a retrospective study conducted on hospitalised adults with CAP (Gadsby et al., 2016). As there are some differences in the aetiology, specimen collection, and antibiotic usage between children and adults, our study can specifically provide evidence for the diagnosis and management of childhood CAP.

It is thought that viruses play a more important role in childhood CAP, and in most studies, the identification rates of viruses are higher than those of bacteria (Johnson et al., 2008; Holter et al., 2015; Jain et al., 2015). However, the opposite was true in our study. *H. influenzae* and *S. pneumoniae* in particular ranked second and third most common pathogens, respectively, with identification rates over 20%. This detection rate was higher than in studies where bacteria were detected using culture and similar to studies that used molecular methods (Elemraid et al., 2013; Howie et al., 2014; Gadsby et al., 2016). Therefore, the detection of bacterial pathogens solely based on culture is difficult to comprehensively reveal the underlying causative pathogen spectrum, which would possibly result in underestimating the risk of bacterial infection following primary viral infection. Multiple-target detection techniques based on real-time PCR and metagenomic next-generation sequencing (mNGS), which have the ability to identify both viral and bacterial infections, can serve as quantitative and comprehensive means of diagnosis, and are ideal for prescribing proper treatment (Gadsby et al., 2015; Miao et al., 2018).

In China, pneumococcal conjugated vaccine (PCV) and *Haemophilus Influenzae* Type b (Hib) were introduced for private purchase against 13 serotypes of *S. pneumoniae* in 2008 and *H. influenzae* type B in 2000 respectively (Wagner et al., 2014). However, these two vaccines were not included in the national immunization program in China, and the proportions of age-appropriate vaccination coverage were critically low (Qu et al., 2017). In the present study, *S. pneumoniae* and *H. influenzae* have been shown the rather high detection rate in children with CAP of Beijing and Hebei. Taking into account the above situation, incorporating PCV and Hib into the free immunization program in China would undoubtedly reduce the prevalence of these two agents and might play important roles in preventing childhood CAP.

Sputum samples are likely to be contaminated with upper airway flora, which might lead to false identification of the true pathogen. This is more likely to occur in patients taking antibiotics because antibiotics will inhibit the growth of the causative bacteria (Rodrigues and Groves, 2018). We speculate that this may be the reason why the results of culture were inconsistent with combined-MS methods in five specimens in this study. For other culture-positive samples, bacteria identified using culture were identical to those identified using combined-MS methods, which set a threshold as previously published for excluding the interference of bacteria colonizing (Gadsby et al., 2016). We therefore suggest that collecting induced sputum in children with CAP and analysing them with quantitative molecular bacterial testing can serve as an important complement to traditional culture methods.

By comparing the initial empirical antibiotic treatment with the pathogen-directed therapy guided by combined-MS methods, we found some useful evidence supporting the use of empirical antibiotics. First, *H. influenzae* was the most commonly identified bacterial pathogen in our study. β-lactam/β-lactamase inhibitor combinations are recommended as its first-line treatment. Therefore, it is recommended the original empirical therapy to be β-lactam/β-lactamase inhibitor combinations in most cases. Second, macrolides are frequently prescribed to children with CAP because of their long half-life and short duration of therapy (Blyth and Gerber, 2018). However, except for CAP caused by atypical pathogens, most macrolides are unnecessary, which was also the case in our study (25.4%) (Al-Niemat et al., 2014; Wunderink, 2018). Overuse of macrolides lead to an economic burden and an increase in macrolide resistance in respiratory pathogens (Gandra et al., 2014; Xiao et al., 2016; McEwen and Collignon, 2018). It is noteworthy that antimicrobial resistance is a crucial consideration when selecting antimicrobials. As the phenotypic characteristics, such as susceptibility, can only be identified using culture-based methods, which offers it irreplaceable advantages over molecular testing. A limitation of the combined-MS method described here was that it cannot provide information on antimicrobial resistance during bacteria detection. However, for multiplex PCR-MS platforms, screening multiple mutations associated with drug resistance is reasonably practicable. Therefore, in the follow-up study, an extra panel targeting resistance genes in key pathogens causative of bacterial pneumonia can be designed and included to complement the detection panel. Combing the positive microbiological identification and AMR profiles of the causative agents, empirical selection of antibiotics would be more effective and individualized.

## Data Availability Statement

The raw data supporting the conclusions of this article will be made available by the authors, without undue reservation.

## Ethics Statement

This study was approved by Medical Ethics Committee of Beijing Children’s Hospital, Capital Medical University. Written informed consent to participate in this study was provided by the participants’ legal guardian/next of kin.

## Author Contributions

JP contributed to the development of the study design and the coordination of the execution of the study. ZX and JP coordinated the study and reviewed drafts of the manuscript. LS and CZ drafted the study protocol, analyzed the results, and drafted the manuscript. SA conducted to the experiment and assisted in writing the manuscript. XC, YL, LX, and BX helped to performed the experiments. All authors contributed to the article and approved the submitted version.

## Funding

This work was funded by the Non-profit Central Research Institute Fund of Chinese Academy of Medical Sciences (2018PT51009, 2019PT310006 and 2019PT310029) and Key Technology R&D Program of China (2017 ZX10103004-004).

## Conflict of Interest

The authors declare that the research was conducted in the absence of any commercial or financial relationships that could be construed as a potential conflict of interest.
