# Supplementary material for: Comprehensive Description of Pathogens and Antibiotic Treatment Guidance in Children With Community-Acquired Pneumonia Using Combined Mass Spectrometry Methods
Source: Front Cell Infect Microbiol. 2021 Jul 21;11:695134. doi: 10.3389/fcimb.2021.695134 (PMC8335481; doi:10.3389/fcimb.2021.695134)
Supplement: Supplementary file 1 [file DataSheet_1.docx]

**Supplementary Material**

**Comprehensive Description of Pathogens and Antibiotic Treatment Guidance in Children with Community-acquired Pneumonia Using Combined Mass Spectrometry Methods**

**Liying Sun,^1,^****^2,†^ Chi Zhang,^1,2,†^ Shuhua An,^3,†^ Xiangpeng Chen,^4^ Yamei Li,^1,2^ Leshan Xiu,^1,2^ Baoping Xu,^5^ Zhengde Xie,^4,*^ Junping Peng,^1,2,*^**

^1^NHC Key Laboratory of Systems Biology of Pathogens, Institute of Pathogen Biology, Chinese Academy of Medical Sciences & Peking Union Medical College, Beijing, China

^2^Key Laboratory of Respiratory Disease Pathogenomics, Chinese Academy of Medical Sciences and Peking Union Medical College, Beijing, China

^3^Hebei Province Children’s Hospital, Shijiazhuang, Hebei 050011, P.R. China

^4^Beijing Key Laboratory of Pediatric Respiratory Infection Diseases, Key Laboratory of Major Diseases in Children, Ministry of Education, National Clinical Research Center for Respiratory Diseases, Research Unit of Critical Infection in Children, Chinese Academy of Medical Sciences, 2019RU016, Laboratory of Infection and Virology, Beijing Pediatric Research Institute, Beijing Children’s Hospital, Capital Medical University, National Center for Children’s Health, Beijing 100045, China

^5^National Clinical Research Center for Respiratory Diseases, Research Unit of Critical Infection in Children, Chinese Academy of Medical Sciences, 2019RU016, Respiratory department, Beijing Children’s Hospital, Capital Medical University, National Center for Children’s Health, Beijing 100045, China

^*^Corresponding author. Junping Peng, NHC Key Laboratory of Systems Biology of Pathogens, Institute of Pathogen Biology, Chinese Academy of Medical Sciences & Peking Union Medical College, No. 6 Rongjing Eastern Street, BDA, Beijing, 100176, China.

Electronic address: [pengjp@hotmail.com](mailto:pengjp@hotmail.com)

Telephone number: 86-10- 67878493.

Fax number: 86-10- 67878493.

^*^Corresponding author. Zhengde Xie, Beijing Key Laboratory of Pediatric Respiratory Infection Diseases, Key Laboratory of Major Diseases in Children, Ministry of Education，National Clinical Research Center for Respiratory Diseases, Research Unit of Critical Infection in Children, Chinese Academy of Medical Sciences, 2019RU016, Laboratory of Infection and Virology, Beijing Pediatric Research Institute, Beijing Children’s Hospital, Capital Medical University, National Center for Children’s Health, Beijing 100045, China.

Electronic address: [xiezhengde@bch.com.cn](mailto:xiezhengde@bch.com.cn)

^†^ Liying Sun, Chi Zhang, and Shuhua An contributed equally to this article.

**Supplementary information**

**Table S1.** Pathogens detected by culture but not selected in combined-MS methods.

**Table S2.** Appropriate antibiotic treatment for bacterial pathogens in children with CAP.

| **Table S1.**  Appropriate antibiotic treatment for bacterial pathogens in children with CAP | |
| --- | --- |
| Bacterial pathogen | Antimicrobial agents ^a^ |
| *Streptococcus pneumoniae* | β-lactam antibiotics (amoxicillin/ceftriaxone) |
| *Haemophilus influenzae* | β-lactam/β-lactamase inhibitor combinations (amoxicillin clavulanic acid/ceftriaxone tazobactam) |
| *Staphylococcus aureus* |  |
| *Moraxella catarrhalis* |  |
| *Escherichia coli* |  |
| *Klebsiella pneumoniae* |  |
| *Pseudomonas aeruginosa* |  |
| *Acinetobacter baumannii* |  |
| No pathogen identified |  |
| *Legionella pneumophila* | Macrolides (azithromycin/erythromycin) |
| *Bordetella pertussis* |  |
| *Mycoplasma pneumoniae* |  |
| *Chamydiae pneumonia* |  |

^a^ According to the Children's Community Pneumonia Diagnosis and Treatment Guidelines (2019 revised) and revised World Health Organization (WHO) Classification and Treatment of Pneumonia in Children at Health Facilities: Evidence Summaries.

| **Table S2.**  Pathogens detected by culture but not selected in combined-MS methods | | |
| --- | --- | --- |
| Culture-based methods | Combined-MS methods | Number of patients |
| *Monilia albica* | *Streptococcus pneumoniae* | 3 |
| *Monilia albica* | *Streptococcus pneumoniae* and *Haemophilus influenzae* | 1 |
| *Monilia albica* and *Klebsiella Pneumoniae* | *Klebsiella Pneumoniae* | 1 |
| *Monilia albica* and *Haemophilus influenzae* | *Haemophilus influenzae* | 1 |
| *Stenotrophomonas maltophilia* | *Streptococcus pneumoniae* | 1 |
| *Streptococcus agalactiae* | bacteria-negative | 1 |
| *Monilia albica* | bacteria-negative | 2 |
